# Unimodular Multi-Input Multi-Output Waveform and Mismatch Filter Design for Saturated Forward Jamming Suppression

**DOI:** 10.3390/s24185884

**Published:** 2024-09-10

**Authors:** Xuan Fang, Dehua Zhao, Liang Zhang

**Affiliations:** 1 National Key Laboratory of Radar Detection and Sensing, Nanjing Research Institute of Electronics Technology, Nanjing 210039, China; fffsk67k@163.com; 2 Nanjing Research Institute of Electronics Technology, Nanjing 210039, China; horsezl@126.com

**Keywords:** electronic countermeasure, interference suppression, MIMO radar, waveform diversity, radar signal processing, pulse compression

## Abstract

Forward jammers replicate and retransmit radar signals back to generate coherent jamming signals and false targets, making anti-jamming an urgent issue in electronic warfare. Jamming transmitters work at saturation to maximize the retransmission power such that only the phase information of the angular waveform at the designated direction of arrival (DOA) is retained. Therefore, amplitude modulation of MIMO radar angular waveforms offers an advantage in combating forward jamming. We address both the design of unimodular MIMO waveforms and their associated mismatch filters to confront mainlobe jamming in this paper. Firstly, we design the MIMO waveforms to maximize the discrepancy between the retransmitted jamming and the spatially synthesized radar signal. We formulate the problem as unconstrained non-linear optimization and solve it using the conjugate gradient method. Particularly, we introduce fast Fourier transform (FFT) to accelerate the numeric calculation of both the objection function and its gradient. Secondly, we design a mismatch filter to further suppress the filtered jamming through convex optimization in polynomial time. The simulation results show that for an eight-element MIMO radar, we are able to reduce the correlation between the angular waveform and saturated forward jamming to −6.8 dB. Exploiting this difference, the mismatch filter can suppress the jamming peak by 19 dB at the cost of an SNR loss of less than 2 dB.

## 1. Introduction

Modern radars face increasing challenges due to the rapid development of electronic attacks (EAs) in electronic warfare [1,2,3]. In order to protect the radar systems in electronic countermeasures (ECMs), engineers have devised various strategies to counteract increasing jamming threats [4,5,6,7,8].

Forward jamming employing Digital Radio Frequency Memory (DRFM) technology represents a significant EA technique [9,10]. DRFM-based forward jammers work by repeating the intercepted signals. These jammers operate in two modes: full-pulse forward jamming [11] and interrupted-sampling repeater jamming (ISRJ) [12]. In full-pulse mode, the jammer intercepts and forwards the entire transmitted signal. ISRJ rapidly samples multiple signal slices within the same pulse repetition interval (PRI) and generates different types of repeating jamming through modulation. Due to the coherence of the forward jamming, it can obtain the gain of matched filtering and coherent integration. Forward jammers can freely modulate the delay and Doppler of the forward jamming, deceiving radar systems to detect false targets in different range cells. When the forward jammer is located in the radar illumination direction, coherent forward jamming in the mainlobe is difficult to eliminate using cancellation techniques [13]. In particular, forward jamming may have a higher power, completely masking the true targets.

Some effective strategies have been proposed to combat forward jamming, such as multistatic radar systems [14,15,16], pulse agility [17,18,19,20] and waveform and filter design [21,22,23,24]. Multi-radar system can use the target spatial–temporal association between multiple radars to reject repeated jamming. For example, Wang uses the Euclidean distance between targets and multi-station radars to identify false targets [14]. However, data cooperation in multi-radar systems is still a challenge. Pulse agility is another common strategy. Switching the transmitted waveform using special mechanisms, the target echo differs from the time-delayed jamming signal, which increases the difficulty of successful deception with forward jamming. Akhtar [19] proposes an improved frequency agile waveform transmission structure to further reduce the PRI by utilizing sparse reconstruction of overlapping waveforms in the time domain. The pulse agility technique still has some disadvantages: it increases the complexity of the subsequent signal processing and reduces the target echo accumulation time. For radar systems in the search period, this shortcoming cannot be ignored. And this method also relies on the strict assumption that the delay in the forward jamming lags by at least one PRI [25]. These methods are unable to combat forward jamming in the same PRI. Jamming signal distortion is the premise of filter design, so waveform design is often simultaneously used to increase the difference between the signal and the jamming [21]. The joint design of intra-pulse waveforms and filters is used to effectively reduce the pulse compression output of ISRJ signals [26]. However, for full-pulse forward jamming signals, as the quantization level of DRFM improves and its phase accuracy increases, the spectral difference gradually diminishes [27], reducing the effectiveness of traditional radar waveform designs.

Multi-input multi-output (MIMO) radar with waveform diversity brings new strategies [28,29,30]. Compared with phased array (PA) radar, MIMO radar searches for targets with a wide beam, which reduces the signal interception probability by decreasing the average spatial energy. In search processing, MIMO radar employs a longer beam dwell time to compensate for the low beam gain. With waveform diversity, MIMO radar has more degrees of freedom (DoFs) to reject jamming. Based on frequency diverse array (FDA)–MIMO radar, Lan [31] used the difference when receiving the angular frequency between the target echo and the jamming signal to reduce time delay false targets. A blind source separation algorithm is used to separate the real echo and the jamming signals of orthogonal frequency division multiplexing (OFDM)–linear frequency modulation (LFM) MIMO radar [32]. Compared with frequency-modulated signals, coded signals have a larger bandwidth and are more challenging for jammers to detect. Zhang [33] proposes a space–time complex modulation method to encode both the carrier frequency and the initial phase of each pulse to suppress ISRJ. The strategy of FDA-MIMO is extended to Element-Pulse Coding (EPC)–MIMO in [34], and the phase increment between neighboring pulses within the same coherent processing interval (CPI) is employed to suppress range-deceptive targets. However, these techniques cannot reject full-pulse forward jamming within the same PRI.

DRFM-based forward jamming exhibits the following characteristics: (i) The transmitting amplifier of the jammer operates in saturation mode to achieve the maximum power. (ii) The ECM device can accurately analyze the intercepted signal, and the forward jamming signal has the same phase as the intercepted signal. For radar signals, saturated forward jamming signal is a coherent signal with a constant modulus, losing its amplitude information. This modulation distortion remains indistinguishable from PA radar. PA radar uses a saturated power amplifier to enhance the detection performance as well, but MIMO radar is not the same as PA radar. The MIMO angular waveform intercepted by the ECM is synthesized through the coherent superposition of diverse waveforms from different channels. Even if the signals in each channel have a constant modulus, the angular waveform still exhibits amplitude envelope fluctuations. As a result, the jamming signals, lacking amplitude information, differ from MIMO angular waveforms. Xu utilizes the amplitude fluctuations in the angular waveform to reduce the peak cross-correlation level (PCCL) between the forward jamming and the angular waveform [35]. However, it fails to consider the relationship between waveform differences and the zero-lag peak. As a result, forward jamming can still obtain filtering gain and produce a false target with zero lag.

**Figure 1:**
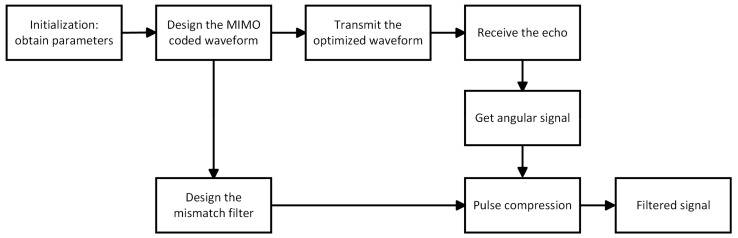
Flowchart of waveform and mismatch filter design.

We propose a scheme that utilizes code division multiple access (CDMA) MIMO radar waveforms combined with a mismatch filter to suppress saturated forward jamming. The implementation of jamming suppression is shown in the flowchart in Figure 1. We use the parameter information obtained to design a MIMO waveform which primarily aims to maximize the amplitude envelope difference between the forward jamming signal and the MIMO angular waveform. After obtaining the optimized waveform, we design a mismatch filter for its angular signal. Saturated forward jamming retains the phase information of the angular signal, but the amplitude difference in the waveforms can be exploited by the MMF to disrupt the correlation between the filter and the jamming signal, thus suppressing the forward jamming.

Our paper makes the following contributions:

i. This paper proposes a strategy for suppressing mainlobe saturated forward jamming using MIMO angular waveform amplitude sequence fluctuations. We propose two waveform design schemes to enhance the signal differences. The first scheme uses the weighted jamming cross-correlation sidelobe level (JCSL) as the criterion. And the second scheme uses the cross-correlation coefficient as the criterion.
ii. The MIMO anti-jamming waveform design is formulated as a multi-objective optimization problem, including the JCSL and the integrated sidelobe level (ISL) of the angular waveform. We solve this non-linear non-convex optimization problem using the conjugate gradient method (CGM). Furthermore, we introduce fast Fourier transform (FFT) to accelerate the calculation of the objective function and its gradient.
iii. The mismatch filter we design can suppress forward jamming by utilizing the amplitude envelope difference. Our filter design is a convex optimization problem that can be solved in polynomial time. For optimized waveforms, the mismatch filter can suppress the jamming peak by 19 dB at the cost of an SNR loss of less than 2 dB while controlling the range sidelobe level.

The remainder of this paper is organized as follows. Section 2 introduces the models for the MIMO radar echo, the jamming signal, and the mismatch filter. In Section 3, we define two anti-jamming waveform design criteria and present fast implementation methods for their objective functions and gradients. In Section 4, we discuss the mismatch filter design criteria for anti-jamming purposes. Section 5 gives some numeric simulation results. The conclusions are presented in Section 6.

**Notation**: We use (·)*, (·)^T^ and (·)^H^ to denote the conjugate, the transpose and the Hermitian transpose. $\left\|\cdot \right\|$, $ℜ\left(\cdot \right)$, $ℑ\left(\cdot \right)$, ∘ and ∇ denote the Frobenius norm, the real part of the argument, the imaginary part of argument, the Hadamard product and gradient operations. vec(·) stands for column-wise vectorization, arg(·) stands for the phase angle of the complex value and diag(·) creates a diagonal matrix with the diagonal elements from a vector. δ_L_ stands for the Kronecker delta function of the time series *L*. **1***_L_* denotes the all-one *L* element column vector, and **I***_L_* denotes the *L* × *L* identity matrix.

## 2. Signal Models

### 2.1. The MIMO Angular Waveform

Consider a co-located MIMO radar system in a target search period which has *N*_t_ transmitting antennas and *N*_r_ receiving antennas. Let $x_{m}\in {\mathbb{C}}^{N_{s}\times 1}$ be the unimodular code sequence of the transmitted signal from the *m*th antenna, which can be defined as

(1) $$s_{m}(t)=\sqrt{\frac{E_{t}}{N_{s}T_{P}}}\sum_{n=1}^{N_{s}}x_{mn}rect(\frac{t-nt_{b}}{t_{b}})\exp(j2\pi f_{0}t)$$

where the unimodular code *x_mn_* = exp(*jφ_mn_*) is the *n*th element of **x***_m_*. *N_s_* is the code length of the transmitted signal. The transmitted code matrix and its phase matrix can be written as $X=[x_{1},x_{2},\ldots ,x_{N_{t}}]$ and $\Phi =[\varphi_{1},\varphi_{2},\ldots ,\varphi_{N_{t}}]$, where $X,\Phi \in {\mathbb{C}}^{N_{s}\times N_{t}}$. Assume that the signal carrier frequency is *f*_0_. We use *rect* (*t*) to produce a rectangular pulse, *t*_b_ is the duration of the unit code and the total time of the impulse signal is *T*_P_ = *N*_s_*t*_b_. The total transmitted energy launched by MIMO is *E*_t_. The amplitude of the sub-signal is defined as χ = $\sqrt{E_{t}/N_{s}T_{P}}$.The transmitting signal is kept at a constant modulus such that the antenna amplifier works in a saturated state to avoid the phase distortion caused by non-linear power amplifiers [36].

The cross-correlation between the pulse signals of the *u*th antenna and the *v*th antenna can be written as

(2) $$r_{uv}(\tau )=\int_{-\infty }^{\infty }s_{u}(t)s_{v}^{*}(t+\tau )d\tau .$$

The cross-correlation of continuous coding signals can be replaced with a discrete coding sequence of cross-correlation form [37]. Thus, the above equation can be rebuilt as

(3) $$r_{uv}[n]=\sum_{i=-\infty }^{\infty }x_{u}[i]x_{v}^{*}[i-n].$$

**Figure 2:**
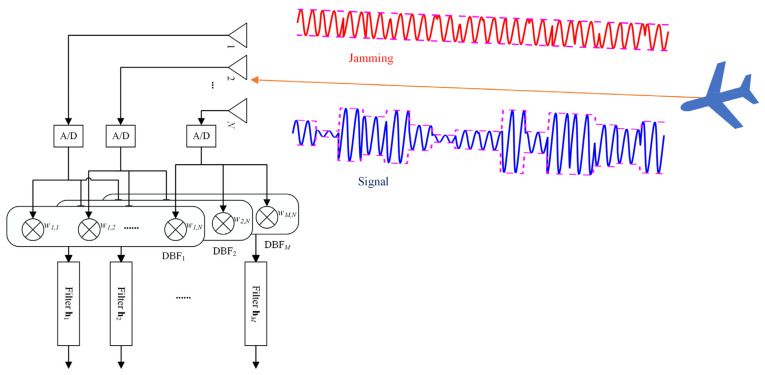
Signal reception and processing model for co-located MIMO radar with spatial synthesis of the waveform and a saturated forward jamming signal in the mainlobe.

To simplify our model in this paper, both the transmitting and receiving antennas are the same uniform linear array (ULA) with half-wavelength array spacing. The receiving signal model is shown in Figure 2. Spatial synthesis of the waveform of the echo in the prescribed direction *θ*, which is called the angular waveform, can be expressed as [38]

(4) $$g_{\theta }=Xa_{t}^{H}(\theta )={\left[g_{\theta }(1),\ldots ,g_{\theta }(N_{s})\right]}^{T},$$

where **a**_t_*(θ)* is the transmitting steering vector and is defined as

(5) $$a_{t}(\theta )=\;[exp\left(j0\psi \right),\ldots ,exp\left(j\left(N_{t}-1\right)\psi \right)].$$

Here, the wave path difference *ψ = 2πd*sin(*θ*)*/λ* is determined by the antenna structure, and in this paper, *ψ = π*sin(*θ*). Due to the waveform diversity in MIMO radar, the amplitude envelope of the angular waveform fluctuates and usually is not a constant-modulus sequence. The cross-correlation between angular waves needs to be limited to avoid spatial sidelobe clutter [35]. The MIMO transmit beam pattern can be modulated by waveform diversity code [38,39], which satisfies the low-intercept requirements.

For MIMO radar, the angular waveform is not fixed. We define a diagonal matrix as

(6) $$D(\theta ,\Delta \theta )=\left[\begin{array}{cccc} \exp\left(j0\tilde{\psi }(\theta ,\Delta \theta )\right) & & & \\ & \exp\left(j\tilde{\psi }(\theta ,\Delta \theta )\right) & & \\ & & \ddots & \\ & & & \exp\left(j\left(N_{t}-1\right)\tilde{\psi }(\theta ,\Delta \theta )\right) \end{array}\right].$$

Δ*θ* is the deflection angle, where

(7) $$\tilde{\psi }(\theta ,\Delta \theta )=2\pi \frac{d}{\lambda }(sin\left(\theta +\Delta \theta \right)-sin\left(\theta \right))+2n\pi ,n\in \mathbb{Z}.$$

The matrix **D**(*θ*, Δ*θ*) is called a deflection matrix. Using the deflection matrix, we can adjust the angular waveform in the θ direction to the *θ* + Δ*θ* direction. The adjusted angular waveform can be written as

(8) $${\tilde{g}}_{\theta +\Delta \theta }=XD(\theta ,\Delta \theta )a_{t}^{H}(\theta +\Delta \theta )=g_{\theta }.$$

In this paper, the cross-correlation between the angular waveforms is not considered. We use digital beamforming (DBF) in the receiving antennas to isolate the angular signals so that more DoFs can be used to reject the jamming. The echo processing is shown in Figure 2. Using (3), the autocorrelation of the angular signal at *θ* can also be presented as a discrete sequence **r***_θ_ =* [*r_θ_*[1 − *N*_s_],…, *r_θ_*[*N*_s_ − 1]]*^T^*, where the *k*th shift element *r_θ_*[*k*] can be expressed as

(9) $$r_{\theta }[k]=\frac{g_{\theta }^{H}J_{k}g_{\theta }}{g_{\theta }^{H}g_{\theta }}=\frac{a_{t}(\theta )X^{H}J_{k}Xa_{t}^{H}(\theta )}{a_{t}(\theta )Ra_{t}^{H}(\theta )},1-N_{s}\leq \;k\;\leq \;N_{s}-1.$$

Here, **J***_k_* is the shift matrix and is defined as

(10) $$J_{k}=\left[\begin{array}{cc} 0_{N_{s}-k\times k} & I_{N_{s}-k}\\ 0_{k\times k} & 0_{k\times N_{s}-k} \end{array}\right]=J_{-k}^{H}.$$

The code covariance matrix **R** = **X***^H^***X**. Note that *E_θ_ =* $g_{\theta }^{H}g_{\theta }$ represents the wave energy at *θ*, which is used for range sidelobe normalization to forsake the null solutions that *E_θ_ = 0*. The elements in **r***_θ_* are conjugate symmetric, i.e., $r_{\theta }^{*}[k]=r_{\theta }[-k]$.

### 2.2. Saturated Forward Jamming Signals

Consider the target in direction *θ* with a DRFM-based forward jammer. The jamming style is shown in Figure 2. The jammer intercepts and repeats the entire pulse signal. In order to generate the jamming signal with the same phase as the echo, the amplifier of the jammer operates in a saturated state. Therefore, the jamming signal undergoes amplitude modulation and usually maintains a constant modulus while losing the amplitude envelope information [27]. The forward jamming signal $s_{jam}(\theta )\in {\mathbb{C}}^{N_{s}}$, which is highly coherent with the echo, can be expressed as follows.

(11) $$s_{\mathrm{jam}}(\theta )=\eta y_{\theta }=\eta \exp(j\kappa (\theta )),$$

where *η* represents the jamming intensity relative to the single-channel signal *s_m_*(*t*), the unimodular jamming waveform $y_{\theta }=\exp(j\kappa (\theta ))$, and $\kappa (\theta )=\arg(g_{\theta })$ extracts the phase of the angular wave. Mathematically, it can also be expressed as

(12) $$y_{\theta }[k]=\frac{g_{\theta }[k]}{\left|g_{\theta }[k]\right|}.$$

For this constant-modulus signal, the total energy *E*_jam_ = $\eta^{2}N_{s}$, and the signal-to-jamming ratio (SJR) for the angular signal is $E_{\theta }/E_{jam}$. The cross-correlation of the angular waveform and jamming can be presented as a discrete sequence **r**_j,*θ*_ *=* [*r*_j,*θ*_[1 − *N*_s_],*…*, *r*_j,*θ*_[*N*_s_ − 1]]*^T^*, where the *k*th shift element *r*_j,*θ*_[*k*] can be expressed as

(13) $$r_{j,\theta }[k]=\eta \frac{g_{\theta }^{H}J_{k}y_{\theta }}{g_{\theta }^{H}g_{\theta }}=\eta \frac{a_{t}(\theta )X^{H}J_{k}y_{\theta }}{a_{t}(\theta )Ra_{t}^{H}(\theta )}.$$

The element *r*_j,*θ*_[*k*] is also normalized with *E_θ_*, and the result is equal to the matched filter output. This element is non-linear; therefore, the function composed of this element is also non-linear.

If the forward jamming is generated in the same PRI as the intercepted signal, false targets can only be generated at the positive offset of the jammer range bin. If they are in different periods, false targets may be generated at both positive and negative range offset positions. Using pulse agility design [19], PA radar can solve the second case effectively. Although the forward jamming signal is amplitude-modulated, the PA radar signal typically has a constant modulus; thus, we cannot use the amplitude difference to suppress the jamming signal. For MIMO radar systems, we can leverage the amplitude envelope fluctuations to enhance the differentiation between angular waves and forward jamming signals, forming the basis for jamming suppression.

### 2.3. Mismatch Filters

When the forward jamming signal and the angular wave have different amplitude envelopes, their phase similarity allows the jamming signal to acquire matched filtering gain. To reduce the pulse compression gain and suppress the jamming signal, we consider designing a mismatch filter. The mismatch filter $h\in {\mathbb{C}}^{L\times 1}$ is shown as

(14) $$h={\left[h(1),h(2),\ldots h(L)\right]}^{T},$$

where *L* is the filter length. In this paper, we assume *L = N_s_* so that the filter length is the same as the code length. Under this assumption, the pulse compression output of the angular waveform and the jamming signal at the *k*th shift can, respectively, be expressed as

(15) $$r_{s,h}[k]=h^{H}J_{k}g_{\theta },$$

(16) $$r_{j,h}[k]=h^{H}J_{k}s_{\mathrm{jam}}(\theta )=\eta h^{H}J_{k}y_{\theta },$$

where 1 − *N_s_ ≤ k ≤ N_s_* − 1. In order to attain a higher SNR and reduce the complexity of the signal model, we design mismatch filters specifically for angular waveforms rather than diverse MIMO waveforms. Since the angular waveform and the forward jamming employ the same phase, suppressing the forward jamming with a mismatch filter will reduce the output energy of the echoes, leading to loss of the SNR. This enhances the impact of noise on the signal. We consider the SNR loss as a metric for mismatch filter design.

Joint optimization of the waveform and filter is a good choice. However, the waveform optimization problem considered in this paper is a non-linear non-convex problem. Joint optimization may increase the complexity of the algorithm. We consider waveform design and filter design as two separate problems following the flowchart in Figure 1. We first design the waveform to maximize the difference between the angular waveform and the forward jamming and then design the mismatch filter for the optimized waveform. The criteria for waveform design are considered in Section 3. The specific details of the filter design are discussed in Section 4.

## 3. Design Formulations for MIMO Waveforms

Several factors are jointly taken into account when designing the anti-jamming waveform. Firstly, we want to decrease the range sidelobe level of the angular waveform in the target’s direction. Secondly, we try to reduce the jamming cross-correlation sidelobe level to reject jamming. Finally, we expect to reduce the correlation between the angular waveform and the jamming signal.

### 3.1. Range Sidelobe Suppression for Angular Waveform

Neighboring strong target echoes may overwhelm the weak target echoes; thus, the angular waveform should avoid sidelobes with high autocorrelation. We calculate the ISL of the angular waveform as the metric of the range sidelobe level.

(17) $$ISL(\theta )=\sum_{k=1-N_{s}}^{N_{s}-1}{\left|r_{\theta }[k]\right|}^{2}-{\left|r_{\theta }[0]\right|}^{2}=r_{\theta }^{H}r_{\theta }-1.$$

From (9), each element has been normalized; thus, the zero lag element *r_θ_*[0] = 1. In this case, the elements of the autocorrelation ambiguity function can be regarded as the matched filtering output.

### 3.2. Mainlobe Jamming Suppression

The target echo is attenuated twice through the emission-reflection processing, while the jamming signal is only attenuated once. The power of the forward jamming signal is higher than that of the echo until it reaches the burn-through distance. We aim to minimize the impact of strong jamming on the signal processing. Therefore, we need to reduce the received jamming energy. We use *JCSL*(*θ*) as the metric for the received jamming energy.

(18) $$JCSL(\theta )=\sum_{k=1-N_{s}}^{N_{s}-1}{\left|r_{j,\theta }[k]\right|}^{2}=r_{j,\theta }^{H}r_{j,\theta }.$$

Like (17), the above formula has been normalized, and the components of *JCSL*(*θ*) correspond to the outputs of the matched filter. The output energy of the matched filter essentially reflects the correlation between the jamming signal and the angular waveform at different shifts.

### 3.3. Enhancement of the Waveform Difference

Saturated forward jamming precisely preserves the true phase of the angular waveform, allowing it to benefit from signal processing. For full-pulse forward jamming, the maximum filtering gain is achieved at zero shift, which is commonly referred to as the mainlobe. Consequently, it is crucial to minimize the high gain obtained to prevent false targets. The key to reducing the filtering gain lies in increasing the waveform difference, which translates to enhancing the amplitude fluctuations in the angular waveform.

We consider two methods for mainlobe suppression. In the first one, we use a cost function to suppress the *0*^th^-lag jamming cross-correlation level (JCL), which is defined as

(19) $$JCL(\theta )={\left|r_{j,\theta }[0]\right|}^{2}.$$

So, we can reformulate (18) into a novel expression.

(20) $$JCSL(\theta ,w)=\sum_{k=1-N_{s}}^{N_{s}-1}w_{k}{\left|r_{j,\theta }[k]\right|}^{2}=r_{j,\theta }^{H}\Lambda r_{j,\theta }.$$

where **Λ** = *diag*([*w*_1*−N*s_,…, *w_−_*_1_, *w*_0_, *w*_1_,…,*w_N_*_s*−*1_]) is derived by weight sequence **w** = [*w*_1_*_−__N_*_s_, *…*, *w_N_*_s−1_]*^T^*. The sidelobe levels at different shifts can be controlled by the vector **w**. But in this paper, we only consider mainlobe suppression, so **w** is set as *w_k_* = 1, ∀ *k* ≠ 0 and *w*_0_ ≫ 1.

The second method is to reduce the cross-correlation coefficient between the jamming and the real echo. The objective function *P*(*θ*), namely representing the jamming cross-correlation coefficient (JCCC), can be shown as below.

(21) $$P(\theta )=\frac{{\left|g_{\theta }^{H}y_{\theta }\right|}^{2}}{{\left\|g_{\theta }\right\|}^{2}{\left\|h\right\|}^{2}},$$

where $y_{\theta }^{H}y_{\theta }=N_{s}$, and the total energy of the unimodular jamming waveform is determined by the encoding length *N*_s_. *P*(*θ*) and *JCL*(*θ*) are associated. If *E_θ_* remains constant, the larger *P*(*θ*) is, the higher *JCL*(*θ*) is.

### 3.4. Problem Formulation for Waveform Design

We need to consider *ISL*(*θ*), *JCSL*(*θ*), and *P*(*θ*) simultaneously when designing the waveform. To address the multi-objective optimization problem, we utilize a weighted cost function to convert these criteria into a composite objective function. Based on the above discussion, we formulate the two waveform design methods as the following constrained optimization problems:

(22) $$\Phi_{1}=\arg\min\Gamma_{1}(\theta ,\Phi ,\lambda ,w)$$

(23) $$\Phi_{2}=\arg\min\Gamma_{2}(\theta ,\Phi ,\lambda )$$

where the objective function $\Gamma_{1}(\theta ,\Phi ,\lambda ,w)$ of the first method with (16) is


(24) $$\Gamma_{1}(\theta ,\Phi ,\lambda ,w)=\lambda_{1}ISL(\theta )+\lambda_{2}JCSL(\theta ,w).$$

And the objective function $\Gamma_{2}(\theta ,\Phi ,\lambda )$ of the second method with (17) is

(25) $$\Gamma_{2}(\theta ,\Phi ,\lambda )=\lambda_{1}ISL(\theta )+\lambda_{2}JCSL(\theta )+\lambda_{3}P(\theta ).$$

The waveform’s DoFs are finite. When designing the waveform, we need to adjust the cost coefficients to balance multiple criteria. For example, the cost coefficients *λ*_1_ and *λ*_2_ are used to balance *ISL*(*θ*) and *JCSL*(*θ*). Let *α* =*λ*_1_/*λ*_2_; we can reduce the range sidelobe level by increasing *α.* However, it is important to note that increasing *α* may result in an enhancement in the jamming energy. Typically, we use a weighted cost function and objective function scaling to keep the two criteria in the same order of magnitude, i.e., *λ*_1_ = *λ*_2_. To differentiate the two methods, we can rewrite the objective functions as below.

(26) $$\Gamma_{1}(\theta ,\Phi ,\lambda ,w)=\lambda_{1}ISL(\theta )+\lambda_{2}JCSL(\theta )+\lambda_{2}(w_{0}-1)JCL(\theta )+0P(\theta ).$$

(27) $$\Gamma_{2}(\theta ,\Phi ,\lambda ,0)=\lambda_{1}ISL(\theta )+\lambda_{2}JCSL(\theta )+0JCL(\theta )+\lambda_{3}P(\theta ).$$

The first method employs a weight *λ*_2_(*w*_0_ − 1) to suppress *JCL*(*θ*), while the second method uses a cost *λ*_3_ to reduce *P*(*θ*) for *JCL*(*θ*) reduction. The former method achieves *JCL*(*θ*) reduction by simultaneously increasing *E_θ_* and decreasing *P*(*θ*), whereas the latter method focuses solely on lowering *P*(*θ*) to mitigate *JCL*(*θ*). Thus, the first method may result in a higher wave energy. Both methods rely on leveraging the DoFs provided by the MIMO waveform diversity. Increasing the number of transmitting antennas augments the DoFs, which enables more effective suppression of forward jamming. However, the practical availability of the transmitting antennas is limited. To further suppress jamming, mismatch filters can be employed.

### 3.5. The Optimization Strategy

We can convert the objective functions (26) and (27) into a general form as follows:

(28) $$\Gamma (\theta ,\Phi ,\lambda ,w)=\lambda_{1}ISL(\theta )+\lambda_{2}JCSL(\theta ,w)+\lambda_{3}P(\theta ).$$

The optimization problem for this objective function is non-linear and non-convex. The DoFs of MIMO waveform optimization are its coding size. Therefore, MIMO waveforms with multiple waveforms and long coding may have very large DoFs (*N*_s_ × *N*_t_). Interpolation and least squares methods cannot quickly and stably solve large-scale non-convex problems. We solve the problem using the CGM, which is very effective for large-scale problems. In this paper, we adopt the Polak–Ribière CGM (PR-CGM) as the search engine [40], which is more robust. To speed up the problem solving, we calculate the involved objective functions and their gradients via FFT in each iteration.

Objective functions and their gradients can be derived using the convolution theorem and FFT. The gradient of the objective function can be expressed as

(29) $$\nabla_{vec{\left(\Phi \right)}^{T}}\Gamma (\theta ,\Phi ,\lambda ,w)=\lambda_{1}\nabla_{vec{\left(\Phi \right)}^{T}}ISL(\theta )+\lambda_{2}\nabla_{vec{\left(\Phi \right)}^{T}}JCSL(\theta ,w)+\lambda_{3}\nabla_{vec{\left(\Phi \right)}^{T}}P(\theta ).$$

Moreover, $vec(\Phi )={\left[\varphi_{1}^{T},\ldots ,\varphi_{N_{t}}^{T}\right]}^{T}$. We can divide this *N*_t_*N*_s_ × 1 vector into **φ**_m_ and compute the gradient separately. In the following, we use *ISL*(*θ*), *JCSL*(*θ*,**w**), and *P*(*θ*) in three parts to introduce how to calculate the objective function and its gradient.

Taking *ISL*(*θ*) as an example, we first construct the zero-padding sequence ${\bar{g}}_{\theta }\in {\mathbb{C}}^{2N_{s}\times 1}$.

(30) $${\bar{g}}_{\theta }={\left[{\bar{g}}_{\theta }(1),\ldots ,{\bar{g}}_{\theta }(2N_{s})\right]}^{T}={\left[g_{\theta }^{T},0_{1\times N_{s}}\right]}^{T}.$$

Then, we can obtain the 2*N*_s_ point spectrum

(31) $${\bar{G}}_{\theta }=F{\bar{g}}_{\theta },$$

where the 2*N*_s_ order FFT matrix $F\in {\mathbb{C}}^{2N_{s}\times 2N_{s}}$. The element of **F** is, i.e.,

(32) $$F_{i,j}=\exp(-j\frac{2\pi }{2N_{s}}(i-1)(j-1)),1\leq i,j\leq 2N_{s}.$$

According to the convolution theorem, the autocorrelation sequence of the angular signal can be obtained through frequency domain calculation as follows.

(33) $$\begin{array}{cc} {\bar{r}}_{\theta } & =\frac{1}{2N_{s}E_{\theta }}F^{H}({\bar{G}}_{\theta }^{*}\circ {\bar{G}}_{\theta })\\ & =[r_{\theta }(0),r_{\theta }(1),\ldots ,r_{\theta }(N_{s}-1),0,r_{\theta }(1-N_{s}),\ldots ,r_{\theta }(-1){]}^{T}. \end{array}$$

${\bar{r}}_{\theta }\in {\mathbb{C}}^{2N_{s}\times 1}$ is the shift version of **r***_θ_* with zero padding. We define the spectral power density as $P_{f}(\theta )={\bar{G}}_{\theta }^{*}\circ {\bar{G}}_{\theta }$. Then, we can rewrite (17) as

(34) $$\begin{array}{cc} ISL(\theta ) & ={\bar{r}}_{\theta }^{H}{\bar{r}}_{\theta }-1\\ & =\frac{1}{2N_{s}E_{\theta }^{2}}({\bar{G}}_{\theta }^{*}\circ {\bar{G}}_{\theta }{)}^{H}({\bar{G}}_{\theta }^{*}\circ {\bar{G}}_{\theta })-1\\ & =\frac{1}{2N_{s}E_{\theta }^{2}}P_{f}^{H}(\theta )P_{f}(\theta )-1. \end{array}$$

Here, we derive the gradient with **φ**_m_, ∀ m. The gradient of the above function can be transformed into the gradients with respect to (w.r.t) $P_{f}(\theta )$ and *E*_θ_ by the chain rule:

(35) $$\begin{array}{cc} \nabla_{\varphi_{m}^{T}}ISL(\theta ) & =\nabla_{\varphi_{m}^{T}}\frac{P_{f}^{H}(\theta )P_{f}(\theta )}{2N_{s}E_{\theta }^{2}}\\ & =\frac{1}{2N_{s}{\left(E_{\theta }^{2}\right)}^{2}}(E_{\theta }^{2}\nabla_{\varphi_{m}^{T}}P_{f}^{H}(\theta )P_{f}(\theta )-P_{f}^{H}(\theta )P_{f}(\theta )\nabla_{\varphi_{m}^{T}}E_{\theta }^{2})\\ & =\frac{1}{N_{s}{\left(E_{\theta }^{2}\right)}^{2}}(E_{\theta }^{2}ℜ(P_{f}^{H}(\theta )\nabla_{\varphi_{m}^{T}}P_{f}(\theta ))-E_{\theta }P_{f}^{H}(\theta )P_{f}(\theta )\nabla_{\varphi_{m}^{T}}E_{\theta }). \end{array}$$

The gradient function of the left half of (35) w.r.t **P***_f_* (*θ*) can be calculated as

(36) $$\begin{array}{cc} \nabla_{\varphi_{m}^{T}}P_{f}(\theta ) & =\nabla_{\varphi_{m}^{T}}((F_{:,1:N_{s}}g_{\theta }{)}^{*}\circ (F_{:,1:N_{s}}g_{\theta }))\\ & =\nabla_{\varphi_{m}^{T}}(diag((F_{:,1:N_{s}}g_{\theta }{)}^{H})Fg_{\theta })\\ & =2ℜ(diag((F_{:,1:N_{s}}g_{\theta }{)}^{H})F\nabla_{\varphi_{m}^{T}}g_{\theta }). \end{array}$$

Since ${\bar{g}}_{\theta }$ is a zero-padding sequence, ${\bar{G}}_{\theta }=F_{:,1:N_{s}}g_{\theta }$. The partial Fourier matrix $F_{:,1:N_{s}}$ is the first *N*_s_ columns of **F**. The computational complexity can be greatly reduced by using the latter formula.

The gradient function of the right half of (28) w.r.t *E_θ_* can be calculated as

(37) $$\nabla_{\varphi_{m}^{T}}E_{\theta }=2ℜ(g_{\theta }^{H}\nabla_{\varphi_{m}^{T}}g_{\theta }).$$

Thus, the gradients w.r.t **P***_f_* (*θ*) and *E_θ_* are both transformed into the gradient of **g***_θ_*, which can be calculated as

(38) $$\nabla_{\varphi_{m}^{T}}g_{\theta }=1ja_{t,\theta }^{*}\left(m\right)diag(x_{m}).$$

Similarly, **r**_j,*θ*_ in (13) can be transformed with the cross-correlation power density function $C_{f}(\theta )=({\bar{G}}_{\theta }^{*}\circ {\bar{Y}}_{\theta })$ as

(39) $$\begin{array}{cc} {\bar{r}}_{j,\theta } & =\frac{1}{2N_{s}E_{\theta }}F^{H}C_{f}(\theta )\\ & =[r_{j,\theta }(0),r_{j,\theta }(1),\ldots ,r_{j,\theta }(N_{s}-1),0,r_{j,\theta }(1-N_{s}),\ldots ,r_{j,\theta }(-1){]}^{T}. \end{array}$$

Thus, the formula *JCSL*(*θ*) and its gradient can be shown as

(40) $$\begin{array}{cc} JCSL(\theta ,w) & ={\bar{r}}_{j,\theta }^{H}\bar{\Lambda }{\bar{r}}_{j,\theta }\\ & =\frac{1}{{\left(2N_{s}E_{\theta }\right)}^{2}}C_{f}^{H}(\theta )F\bar{\Lambda }F^{H}C_{f}(\theta ), \end{array}$$

(41) $$\nabla_{\varphi_{m}^{T}}JCSL(\theta ,w)=\frac{1}{N_{s}{\left(E_{\theta }^{2}\right)}^{2}}\cdot (E_{\theta }^{2}ℜ(C_{f}^{H}{\left(\theta \right)}^{H}F\bar{\Lambda }F^{H}\nabla_{\varphi_{m}^{T}}C_{f}(\theta ))-E_{\theta }C_{f}^{H}{\left(\theta \right)}^{H}F\bar{\Lambda }F^{H}C_{f}(\theta )\nabla_{\varphi_{m}^{T}}E_{\theta }),$$

where $\bar{\Lambda }$ = *diag*([*w*_0_, *w*_1_,…, *w_N_*_s*−*1_, 0, *w*_1*−N*s_,…, *w_−_*_1_]) is derived by the shift version of vector **Λ**. *JCSL*(*θ*) can be seen as a special case when $\bar{\Lambda }$ = **I**_2*N*s_. The gradient of E*_θ_* is already given in (37). The gradient of **C***_f_* (*θ*) can be derived as

(42) $$\begin{array}{cc} \nabla_{\varphi_{m}^{T}}C_{f}(\theta ) & =\nabla_{\varphi_{m}^{T}}((F_{:,1:N_{s}}g_{\theta }{)}^{*}\circ (F_{:,1:N_{s}}y_{\theta }))\\ & =diag((F_{:,1:N_{s}}g_{\theta }{)}^{H})F\nabla_{\varphi_{m}^{T}}y_{\theta })+diag((F_{:,1:N_{s}}y_{\theta }{)}^{T})F^{*}\nabla_{\varphi_{m}^{T}}g_{\theta }^{*}), \end{array}$$

where the gradients of the vector $y_{\theta }$ and $g_{\theta }^{*}$ are

(43) $$\nabla_{\varphi_{m}^{T}}y_{\theta }=\frac{j}{2}\mathrm{diag}(y_{k})(a_{t,\theta }^{*}(m)\mathrm{diag}(x_{m}){\left(\mathrm{diag}(g_{k})\right)}^{-1}+a_{t,\theta }(m)\mathrm{diag}(x_{m}^{*}){\left(\mathrm{diag}(g_{k}^{*})\right)}^{-1}).$$

(44) $$\nabla_{\varphi_{m}^{T}}g_{\theta }^{*}=-1ja_{t,\theta }\left(m\right)diag(x_{m}^{*}),$$

Finally, we can obtain the gradient of *P*(*θ*) with the chain rule as

(45) $$\begin{array}{cc} \nabla_{\varphi_{m}^{T}}P(\theta ) & =\nabla_{\varphi_{m}^{T}}\frac{{\left|g_{\theta }^{H}y_{\theta }\right|}^{2}}{\left\|g_{\theta }\right\|\left\|y_{\theta }\right\|}\\ & =\frac{1}{N_{s}E_{\theta }^{2}}(E_{\theta }\nabla_{\varphi_{m}^{T}}{\left|g_{\theta }^{H}y_{\theta }\right|}^{2}-{\left|g_{\theta }^{H}y_{\theta }\right|}^{2}\nabla_{\varphi_{m}^{T}}E_{\theta }). \end{array}$$

where

(46) $$\begin{array}{cc} \nabla_{\varphi_{m}^{T}}{\left|g_{\theta }^{H}y_{\theta }\right|}^{2} & =2ℜ((g_{\theta }^{H}y_{\theta }{)}^{H}\nabla_{\varphi_{m}^{T}}(g_{\theta }^{H}y_{\theta }))\\ & =2ℜ((g_{\theta }^{H}y_{\theta }{)}^{H}(g_{\theta }^{H}\nabla_{\varphi_{m}^{T}}y_{\theta }+y_{\theta }^{T}\nabla_{\varphi_{m}^{T}}g_{\theta }^{*})). \end{array}$$

$\nabla_{\varphi_{m}^{T}}E_{\theta }$ is already given in (37). Based on the above discussion, we are able to efficiently calculate the objective function (26), (27) and their associated gradients via FFT. The previous derivations are incorporated into the CGM algorithm in Algorithm 1.
**Algorithm 1:** CGM for Waveform Design**Input:**Initial phase matrix **Φ**^(0)^, *θ*, *η* and iteration index *t* = 0.**Step 1:**g(*θ*) is calculated using (4), and y(*θ*) is calculated using (12).**Step 2:***ISL*(*θ*) and its gradient ∇*ISL*(*θ*) are calculated using (17) and (34); *JCSL*(*θ*,**w**) and its gradient ∇*JCSL*(*θ*,**w**) are calculated using (20) and (40);*P*(*θ*) and its gradient ∇*P*(*θ*) are calculated using (21) and (45).**Step 3:**Γ(**Φ**
^(*t*)^) and its gradient ∇Γ(**Φ**^(*t*)^) are calculated using (28) and (29).**Step 4:**We determine the searching direction ***d****_t_* as $d_{t}=\left\{\begin{array}{l} -\nabla \Gamma (\Phi^{\left(t\right)})\;\;\;\;\;\;\;\;\;\;\;,\;t=0\\ -\nabla \Gamma (\Phi^{\left(t\right)})+\xi_{t}d_{t-1}\;\;\;,\;t>0 \end{array}\right..$
**Step 5:**Find a step-length *ξ_t_* to minimize Γ(**Φ**^(t)^+ *ξ_t_****d****_t_*)**Step 6:**Update the solution as **Φ**^(*t* + 1)^= **Φ**^(*t*)^+ *ξ_t_****d****_t_* and set *t* = *t* + 1.**Step 7:**Return to **Step 1** until the prescribed termination condition is reached.

The CGM is an iterative optimization algorithm. For each iteration, we need to recalculate the objective function and its gradient. If we use numerical methods, the computational complexity of the objective function is 𝒪(*N*^2^), and the complexity of the gradient function is 𝒪(*MN*^3^). After applying FFT acceleration, the complexity of the objective function becomes 𝒪(*N*log_2_*N*), and the complexity of the gradient function becomes 𝒪(*MN*^2^log_2_*N*). This allows us to speed up the computation of large-scale operations.

Through continuous iterative optimization, the CGM algorithm eventually obtains a set of numerical solutions which represent the anti-jamming waveform we want. Their angular waveform has a significant difference in its amplitude envelope fluctuations compared with the saturated forward jamming. However, the saturated forward jamming retains the phase information of the angular waveform, and this coherent signal can still obtain signal processing gain from the matched filtering. We aim to utilize a mismatch filter to eliminate the filtering gain of the jamming signal and further suppress the forward jamming by leveraging the waveform difference.

## 4. Design Formulations for Mismatch Filters

We design the mismatch filter to further suppress the forward jamming. In this paper, we assume the filter length is equal to the coding length. The filter outputs of the phase-encoded waveform are the results of pulse compression. In the pulse compression processing, we aim to achieve three goals. Firstly, we want to reduce the filtering gain of the forward jamming. The second one is to suppress the range sidelobe level of the MIMO angular waveform. Finally, we try to reduce the SNR loss of the designed filter.

### 4.1. Jamming Signal Suppression

As (20), for the pulse compression outputs of the jamming signal, we do not want the jamming sidelobe to cover the true target, nor do we want the high filtering gain obtained at zero lag to generate false targets. To suppress the jamming signal with intensity *η*, we measure the outputs using the weighted filter-jamming cross-correlation integrated energy as

(47) $$J(h,\gamma )=\sum_{k=1-N_{s}}^{N_{s}-1}\gamma_{k}\left|r_{j,h}{\left[k\right]}^{2}\right|,$$

where **γ** = {*γ_k_|*1 *− N*_s_ *≤ k ≤* 1 *− N*_s_} is the weight sequence of the cross-correlation sidelobe level. When *γ_k_* = 1, ∀*k*, *J*(**h**, **γ**) is the actual ISL of the pulse compression outputs. The methods outlined in (24) and (25) exhibit imperfect suppression of jamming at zero lag. To tackle this issue, we propose setting a large value for *γ*_0_ to amplify its weight within the cost function, thereby decreasing the output at zero lag.

We construct the (2*N_s_* − 1) × *N_s_* jamming signal matrix as

(48) $$\hat{Y}={\left[\begin{array}{ccc} 0 & & y_{\theta }(1)\\ & ⋰ & \vdots \\ y_{\theta }(1) & & y_{\theta }(N_{s})\\ \vdots & ⋰ & \\ y_{\theta }(N_{s}) & & 0 \end{array}\right]}_{(2N_{s}-1)\times N_{s}}.$$

Therefore, (39) can be rewritten as

(49) $$\begin{array}{cc} J(h,\gamma ) & =r_{j,h}^{H}diag(\gamma )r_{j,h}\\ & =\eta^{2}h^{T}{\hat{Y}}^{H}diag(\gamma )\hat{Y}h^{*}\\ & =\eta^{2}h^{T}R_{J}h^{*}, \end{array}$$

where the jamming cross-correlation sequence can be calculated as $r_{j,h}=\eta \hat{Y}h^{*}$. The semi-positive definite matrix $R_{J}=R_{J}^{H}$. When *γ_k_* =1 for ∀*k*, $R_{J}$ is presented as the covariance matrix of the jamming signal.

### 4.2. Range Sidelobe Suppression for Mismatch Filter

This criterion is to protect neighboring weak targets. Similar to the waveform design, we use the energy of the sidelobe levels as the objective function. To calculate the range sidelobe level, using (15), we define the objective function as follows [40].

(50) $$I(h)=\sum_{k=1-N_{s}}^{N_{s}-1}{\left|r_{s,h}[k]\right|}^{2}-{\left|r_{s,h}[0]\right|}^{2}.$$

For the above equation, we force the compressed peak *r*_s,h_[0] = 1 to avoid the trivial solution **h** = 0 [41]. Just like for (49), we can rewrite (50) using the echo signal matrix $\hat{G}$ as

(51) $$\begin{array}{cc} I(h) & =h^{T}{\hat{G}}^{H}\hat{G}h^{*}-1\\ & =h^{T}R_{s}h^{*}-1, \end{array}$$

where $\hat{G}$ is defined as

(52) $$\hat{G}={\left[\begin{array}{ccc} 0 & & g_{\theta }(1)\\ & ⋰ & \vdots \\ g_{\theta }(1) & & g_{\theta }(N_{s})\\ \vdots & ⋰ & \\ g_{\theta }(N_{s}) & & 0 \end{array}\right]}_{(2N_{s}-1)\times N_{s}}.$$

The angular waveform covariance matrix $R_{s}=R_{s}^{H}$, which is also a semi-positive definite matrix.

### 4.3. SNR Loss

When designing the mismatch filter, the gain loss of our filter is also crucial to consider. The SNR loss can be defined as

(53) $$SNR_{\mathrm{loss}}=\frac{{\left\|g_{\theta }\right\|}^{2}{\left\|h\right\|}^{2}}{{\left|r_{s,h}[0]\right|}^{2}}.$$

The SNR loss is the expense of the mismatch filter confronting jamming and reducing the signal sidelobes. We propose the metric for the SNR loss as

(54) $$L(h)=h^{H}h.$$

### 4.4. Problem Formulation for Waveform Design

Based on the preceding discussion, we can formulate the multi-objective optimization problem for filter design as the following constrained optimization equation:

(55) $$\begin{array}{l} h=\arg\min\beta_{1}J(h,\gamma )+\beta_{2}I(h)+\beta_{3}L(h)\\ s.t.h^{H}g_{\theta }=1. \end{array}$$

We also utilize a weighted cost function to convert multiple criteria into a composite objective function and then use a single-objective optimization method to solve it. The selection of the cost coefficients keeps these criteria within the same order of magnitude. In fact, the jamming mainlobe suppression problem can also be transformed into a constraint, and the optimization in Equation (55) can be rewritten as follows.

(56) $$\begin{array}{ll} h & =\arg\min h^{T}(\beta_{1}\eta^{2}{\hat{Y}}^{H}\hat{Y}+\beta_{2}{\hat{G}}^{H}\hat{G}+\beta_{3})h^{*}\\ s.t. & h^{H}g_{\theta }=1\\ & {\left|h^{H}y_{\theta }\right|}^{2}\leq \varepsilon . \end{array}$$

Compared with (55), the new optimization equation consumes an extra DoF to suppress the jamming mainlobe. It may bring a large loss in the SNR when the jamming intensity is quite strong or the JCCC is high. The objective function of (47) can be written as

(57) $$\begin{array}{cc} \Gamma_{3}(h,\beta ) & =\beta_{1}J(h,\gamma )+\beta_{2}I(h)+\beta_{3}L(h)\\ & =\beta_{1}\eta^{2}h^{T}R_{J}h^{*}+\beta_{2}(h^{T}R_{s}h^{*}-1)+\beta_{3}h^{T}h^{*}\\ & =h^{T}(\beta_{1}\eta^{2}R_{J}+\beta_{2}R_{s}+\beta_{3})h^{*}-\beta_{2}\\ & =h^{T}R_{\Gamma }h^{*}-\beta_{2} \end{array}$$

Since in (49) and (51) $R_{\Gamma }=\beta_{1}\eta^{2}R_{J}+\beta_{2}R_{s}+\beta_{3}$ and $R_{\Gamma }⪰O$, $R_{\Gamma }$ is a semi-positive definite matrix. Thus, the optimization equation is convex, and we can use the SQP algorithm in the CVX toolbox to solve the problem.

## 5. Numeric Results

In this section, we illustrate the anti-jamming effect of our strategy through numerical simulations. We consider a co-located MIMO radar system with the same ULA antenna for both transmission and reception. The spacing between adjacent antennas is half a wavelength apart, and the radiation pattern of each antenna is omni-directional. During the simulation process, each antenna transmits an independent phase-coded waveform. We assume that the target and the jammer are both located in the prescribed direction of 10°. This angle is not fixed and is only used in the simulations of this section. We first analyze the performance of waveform design algorithms and then discuss the performance of mismatch filters.

### 5.1. Results of the Waveform Design

We first demonstrate the performance of both algorithms with diverse eight-element waveforms. The encoding length *N*_s_ of each waveform is 128, which means the waveform is composed of 128 coherent sub-pulses. We assume that the amplitude χ = 1 V for all sub-signals. The parameter settings for the two methods are shown in Table 1. In this section, “JCL” generally refers to the JCL at zero shift.

The parameters in the table scale each criterion to the same magnitude. The initial values of phase encoding for each experiment are obtained through random numbers, denoted as WF0. The first method (i.e., WF1) discussed in (24) uses the weighted JCSL to suppress the JCL. WF2 represents the approach in (25), which employs a cost function to reduce the JCCC and suppress the JCL.

**Figure 3:**
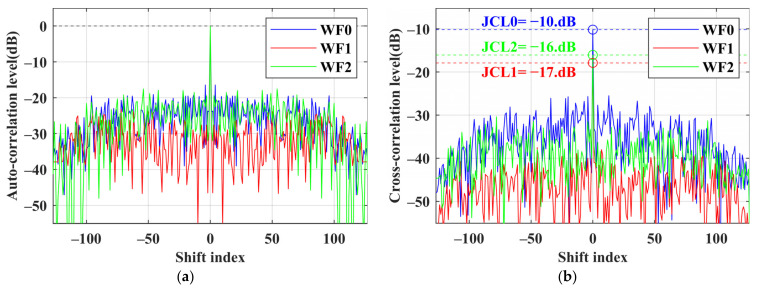
Comparison of waveform performance of each method in 10° for 8 × 128 codes when *η* = 1. (**a**) Autocorrelation level; (**b**) jamming cross-correlation level.

We first assume the jamming intensity *η* = 1, which means the amplitude of the jamming signal is 1 V. To showcase the performance of each method, we present a group of optimized waveforms in Figure 3. Figure 3a,b show the autocorrelation range sidelobe levels and the jamming cross-correlation range sidelobe levels of the three angular waveforms. The blue line represents the performance of WF0. The red line shows that WF1 is the lowest in both figures. The green line, representing WF2, is higher than the red line but lower than the blue line. The numerical results of the three waveforms are presented in Table 2. The JCL of WF0 is −10.1 dB. WF1 exhibits better suppression effects on the JCSL, with only 10% of WF0, while WF2 achieves about 30% of WF0. WF2 reduces the JCL at zero lag to −16.0 dB, and WF1 performs better with a reduction to −17.8 dB. Combined with the JCCC and *E*_θ_ in Table 2, WF1 mainly suppresses the cross-correlation sidelobes by enhancing the waveform energy *E*_θ_, whereas WF2 mainly reduces *P*(*θ*) to decrease the JCL. Both optimization methods effectively suppress the forward jamming signals.

**Figure 4:**
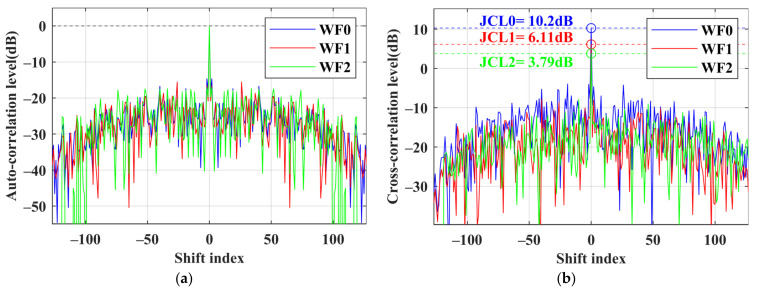
Comparison of waveform performance of each method in 10° for 8 × 128 codes when *η* = 10. (**a**) Autocorrelation level; (**b**) jamming cross-correlation level.

Assuming that the phase of the forward jamming is the same as the real echo, the cross-correlation levels discussed in (13) are also related to the jamming intensity *η*. We present a group of optimized waveforms with the jamming intensity *η* = 10 in Figure 4 to illustrate this. The amplitude of the jamming signal is now 10 V. In Figure 4a, the autocorrelation lines of the three waveforms are mixed together. In Figure 4b, the green line representing WF2 is the lowest, followed by the red line for WF1. At this time, WF2 has a better jamming suppression effect. The numerical results in Table 2 also show this. Compared to Figure 3, the jamming effect on the radar is greatly enhanced due to the increased jamming intensity. The JCL of WF0 is 10.2 dB. While both methods control the JCCC at a level similar to *η* = 1, noticeable differences arise in terms of the JCL and the JCSL. For WF2, the increased jamming intensity has a minor impact, the JCSL of WF2 remains at around 30% of WF0 and the JCL reduces to 3.79 dB. However, for WF1, the increased jamming intensity reduces its performance, with the JCSL being around 40% of WF0, and the JCL only reduces to 6.11 dB. Combined with the JCCC and E*_θ_* in Table 2, the decreased energy E*_θ_* results in performance degradation. Due to the increased weight of the jamming metrics, although both methods still exhibit good suppression effects on jamming, the performance of the autocorrelation range sidelobes has decreased. In practical applications, full recovery of the assumed phase information by the DRFM is unattainable, resulting in lower JCCC values compared with the simulations. Therefore, we may adjust the cost coefficients to suppress the ISL of the synthesized signals appropriately.

**Figure 5:**
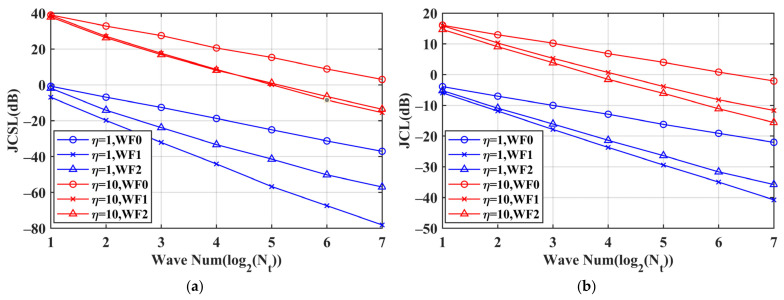
Performance comparison for different wave numbers with code length Ns = 128. (**a**) JCSL of WF0, WF1 and WF2 at different jamming intensities; (**b**) JCL of WF0, WF1 and WF2 at different jamming intensities at zero lag.

As is widely recognized, MIMO radar utilizes waveform diversity to achieve anti-jamming advantages. The more MIMO radar waveforms there are, the more DoFs there are in waveform optimization. This study investigates the impact of the wave number *N*_t_. The performance for each coding length is obtained by averaging the results of 10 repeated experiments, as shown in Figure 5. Combining Figure 5a,b, it becomes evident that under both jamming intensities, the jamming suppression effect improves with increased DoFs. Figure 5a demonstrates that the JCSL decreases as *N*_t_ increases, and Figure 5b shows a decrease in the JCL at zero lag with increasing *N*_t_. Increasing *N*_t_ increases the energy *E*_θ_. Since the JCSL is normalized by *E*_θ_, increasing *E*_θ_ will lead to a decrease in the JCSL. Moreover, increasing *N*_t_ expands the range of amplitude values of the angular waveform, resulting in more drastic amplitude envelope fluctuations, which increase the waveform difference. The lower bound of the cross-correlation coefficient JCCC is 1/*N_t_*, meaning that a higher number of waveforms leads to greater distinguishability between the waveforms. Therefore, the JCL decreases with increasing *N*_t_.

By comparing Figure 3b, Figure 4b and Figure 5, respectively, it is observed that both methods exhibit lower cross-correlation levels compared to WF0. Specifically, WF1 is more effective when *η* is low, and its jamming metrics decrease at a faster rate. As the jamming intensity increases, the performance of WF2 surpasses WF1, and the decline in the jamming metrics is also more pronounced for WF2. When the jamming intensity further increases, the JCSL will be much larger than the ISL, and the target echo will be drowned out by the jamming sidelobes.

We have only considered a single jamming case. For a jammer in any direction, we only need to use a deflection matrix **D**(*θ*, Δ*θ*) to adjust the pre-optimized angular waveform in the direction of jamming. If the number of jamming sources increases, we need to build a large waveform library or optimize the waveform in real time. Although our algorithm can be used in multi-jammer scenarios, we need to accurately acquire the jamming parameters using cognitive waveforms. This will be our future research work.

### 5.2. Results of the Mismatch Filter Design

Combined with the design of a mismatch filter, further suppression of forward jamming is feasible. We verify this proposition by utilizing three waveforms acquired at a jamming intensity *η* = 10. As previously stated, the length *L* of the mismatch filter is equal to the waveform length *N*_s_. In light of *η* = 10, Figure 4b demonstrates that the high-power output occurring at zero lag after the matched filtering of the jamming signal generates a false target. The primary aim at this stage is to effectively suppress the JCL and the JCSL. In mismatch filter design, we use the sequence weight vector ***γ*** to reduce the mainlobe peak. The cost function parameters of the mismatch filter are set to *β*_1_ = 1, *β*_2_ = 1 and *β*_3_ = 1, and the weight vector ***γ*** is set as follows:

(58) $$\gamma_{k}=\left\{\begin{array}{cc} 1 & k\neq 0\\ 10 & k=0 \end{array}\right.$$

**Figure 6:**
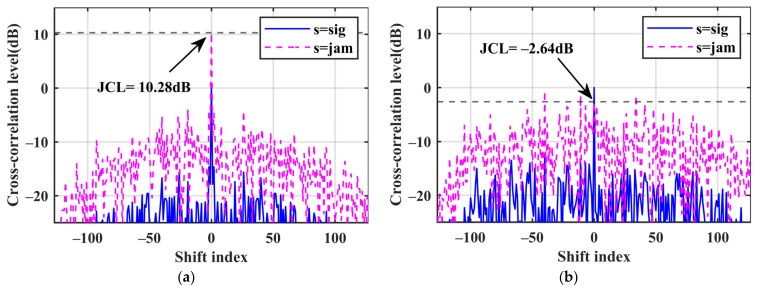
Performance comparison of the two filters for WF0. (**a**) The WF0 matched filter; (**b**) the WF0 mismatch filter.

**Figure 7:**
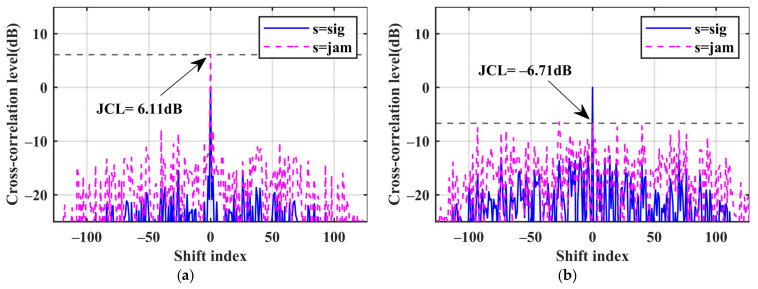
Performance comparison of the two filters for WF1. (**a**)The WF1 matched filter; (**b**) The WF1 mismatch filter.

**Figure 8:**
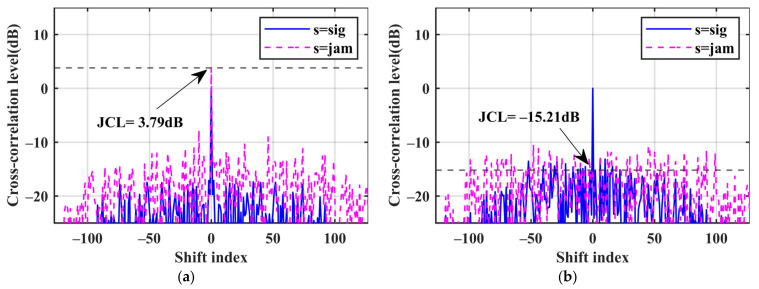
Performance comparison of the two filters for WF2. (**a**) The WF2 matched filter; (**b**) the WF2 mismatch filter.

The matched filter is denoted as MF, and the proposed mismatch filter is denoted as MMF. The pulse compression outputs of the three waves are shown in Figure 6, Figure 7, Figure 8. Table 3 presents the numerical results that we focus on. We can find that the MMF has a good suppression effect on the JCL, and as the cost, the SNR loss is increased.

Figure 6a,b illustrate the two filtered outputs for WF0, where the MMF effectively suppresses the JCL with a degradation of approximately 12.9 dB. This suppression, however, comes at the cost of an approximately 6.8 dB loss in the SNR. Regarding the output of the mismatch filter, radar may not generate false targets. However, it is worth noting that the presence of high-power jamming signals can potentially overshadow the real echoes.

Figure 7a,b show the two filtered outputs of the WF1 waveform, where the JCL is degraded by about 12.8 dB and the SNR loss is about 6.3 dB. For the WF1 waveform, the jamming range sidelobes are no longer able to mask the signal, and the signal coherent accumulation can compensate for the degradation of detection performance caused by the mismatch SNR loss.

Figure 8a,b show the two filter outputs of WF2. The JCL of the MMF is effectively reduced to −15.2 dB, which is a 19 dB decrease compared with the MF. The JCSL of the MMF is 1.56 dB lower than that of the MF, with a minimal SNR loss of 1.97 dB. Compared with Figure 6 and Figure 7, we can find that the MMF designed with WF2 performs the best. The MMFs of the three waveforms constrain the ISL to similar levels. The MMF designed with WF2 achieves the best jamming suppression performance at the lowest SNR loss.

The experimental results demonstrate that the mismatch filter can effectively enhance the jamming suppression performance. When combined with anti-jamming waveform design, more specifically the WF2 method, it yields better jamming suppression.

### 5.3. Application of the Waveform and the Mismatch Filter

To demonstrate the suppression effect of the optimized waveform and the mismatch filter on saturated forward jamming better, a series of point target signal processing examples are given in this section.

Assuming that the point target is in the 100th range bin, the sub-signal amplitude χ = 1 V. To mask the true target, the forward jammers generate the forward jamming signal in five range bins of 50, 150, 200, 250 and 300 with the jamming intensity *η* = 10 V. The coded waveform adopted by the MIMO transmitter in this section is the WF2 waveform with *η* = 10, as shown in Figure 4. The noise power is 10 dBw.

**Figure 9:**
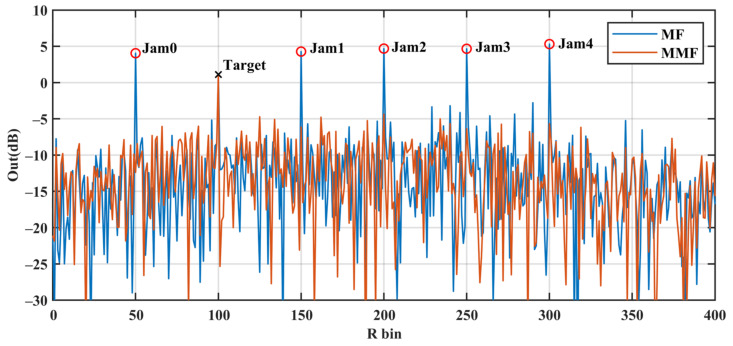
Comparison of pulse compression outputs with the MF and MMF, of which the input signal is composed of a WF2 angular echo at 100 with χ = 1 V, 5 saturated forward jamming bins at 50, 150, 200, 250 and 300 with the jamming intensity *η* = 10 V and Gaussian noise with σ2 = 10 dBw.

The results of pulse compression for the received sampled signal through the MF and the MMF are shown in Figure 9. The signal yielded six peak values after MF processing, five of which were false targets generated by the forward jamming signals. The filtered output of the jamming is at least 2.2 dB higher than that of the real echo. In contrast, only a single peak signal generated by the real echo remains after the signal passes through the MMF, with the other five false target signals suppressed. At this point, the jamming output is at least 6.95 dB lower than the echo. Therefore, our proposed strategy demonstrates effective suppression of forward jamming.

It is worth noting that the angular waveform energy *E*_θ_ is 30.44 dBw, the signal-to jamming ratio (SJR) is −10.56 dB and the input SNR is −0.63 dB. The outputs of the MF and MMF have an SNR of about 20 dB, where the noise is significantly lower than the signal sidelobe in Figure 8. These high sidelobes are caused by the neighboring forward jamming. If the power of the forward jamming is further increased, the jamming sidelobe will overwhelm the real target. We will conduct further research to address this issue.

## 6. Conclusions

Full-pulse forward jamming is highly coherent with real signals and can easily deceive radars into generating false targets. Amplitude modulation of angular waveforms gives MIMO radar an advantage in combating constant-modulus saturated forward jamming. We propose a scheme that utilizes MIMO radar waveforms combined with mismatch filtering to suppress forward jamming in the mainlobe. Two waveform design methods are proposed, quantifying the waveform difference between the angular waveform and the forward jamming with different metrics. To reduce the computational cost of large-scale operations, this paper utilizes the CGM to solve the waveform optimization problem and FFT to accelerate the calculation of the objective function and its gradient in each iteration. To suppress the jamming peak at zero shift, we design a mismatch filter for the optimized waveform. The numerical simulation results demonstrate that our methods can effectively suppress saturated forward jamming. In strong jamming environments, the WF2 scheme exhibits a better performance. Using mismatch filters, we can suppress forward jamming with a small loss in the SNR.

We leverage the waveform diversity of MIMO radar to reject saturated forward jamming. However, if the jamming intensity further increases, the jamming signal may completely overwhelm the true signal. In this situation, the proposed method would be unable to identify real targets. In the future, we will further explore how to suppress strong jamming which can cover signals.

## Figures and Tables

**Table 1:** Parameter settings for two methods.

Wave Type	*λ*_1_	*λ*_2_	*λ*_3_	*w*_0_
WF1	1	1	/	160
WF2	1	1	160	/

**Table 2:** Performance comparison of the three waveforms with different jamming intensities.

Power	Wave Type	ISL	JCSL	JCL (dB)	*P*(θ) (dB)	*E_θ_* (dB)
*η* = 1	WF0	0.93	0.21	−10.17	−1.01	30.23
	WF1	0.25	0.02	−17.87	−1.95	37.00
	WF2	0.90	0.06	−16.05	−6.19	30.93
*η* = 10	WF0	0.92	23.80	10.28	−1.21	29.58
	WF1	0.79	8.72	6.11	−1.40	33.56
	WF2	1.01	6.72	3.79	−6.77	30.44

**Table 3:** Performance comparison of the three waveforms with the MF and MMF.

Filter Type	Wave Type	ISL	JCSL	JCL (dB)	SNR_loss_ (dB)
MF	WF0	0.92	23.80	10.28	0.00
	WF1	0.79	8.72	6.11	0.00
	WF2	1.01	6.72	3.79	0.00
MMF	WF0	2.11	25.89	−2.64	6.84
	WF1	2.23	8.44	−6.71	6.28
	WF2	1.99	4.69	−15.21	1.97

## Data Availability

The data presented in this study are available on request from the corresponding author.
